# Exploring the experiences of women and people with diabetes in pregnancy in metropolitan and rural Australia: a national survey

**DOI:** 10.1186/s12884-024-07093-8

**Published:** 2025-01-08

**Authors:** Ellen Payne, Susan Heaney, Clare Collins, Megan Rollo, Leanne J. Brown

**Affiliations:** 1 School of Health Sciences, College of Health, Medicine and Wellbeing, The University of Newcastle, NSW, Australia; 2 Department of Rural Health, College of Health, Medicine and Wellbeing, University of Newcastle, Tamworth, NSW Australia; 3 Department of Rural Health, College of Health, Medicine and Wellbeing, University of Newcastle, Port Macquarie, Tamworth, NSW Australia; 4 Food and Nutrition Research Program, Hunter Medical Research Institute, New Lambton Heights, NSW Australia; 5 School of Population Health, Faculty of Health Sciences, Curtin University, Bentley, WA Australia

**Keywords:** Rural health, Diabetes in pregnancy, Models of care, Gestational diabetes mellitus, Healthcare delivery

## Abstract

**Background:**

Women and people diagnosed with diabetes in pregnancy, are recommended to have frequent monitoring and careful management for optimal pregnancy outcomes. This health care management should be supported by a multidisciplinary healthcare team. For individuals living in rural areas, there are increased barriers to healthcare access, with subsequent worse health outcomes compared to those in metropolitan regions. Despite this, there remains a lack of research into the experiences of healthcare delivery for rural women and people with diabetes in pregnancy.

**Methods:**

Survey invitations were sent via the National Diabetes Services Scheme email list. The survey included multiple choice and open-ended questions. Responses from the open-ended question asking participants the changes they would want made to their care delivery were interpreted using qualitative content analysis. Responses were separated into metropolitan and rural categories using the Modified Monash Model criteria.

**Results:**

There were 668 survey responses, with 409 responding to the open-ended qualitative survey question/s. 71.6% of respondents were metropolitan and 27.6% lived rurally. A total of 31 codes were established from the open-ended responses, with the five overarching themes of ‘quality of care’, ‘practice & communication’, ‘individual’s experience’, ‘access’ and ‘burden of care’ identified. The most frequently occurring codes irrespective of location included education or information (n = 45), frequency and timeliness of care (n = 42), no changes (n = 42) and improved health professional communication (n = 40). Local care options was the only code with more rural quotes compared to metropolitan.

**Conclusions:**

The most frequently occurring codes had strong representation from metropolitan and rural respondents, indicating that those with previous diabetes in pregnancy had similar priorities for changes in their healthcare delivery regardless of location. Rural respondents identifying local care options as a priority for change is likely indicative of the rural healthcare landscape with limited access to care options. Recommendations from this study supported by previous research include focusing on improving health professional communication both with women and people with diabetes in pregnancy and with other relevant professionals. Recommendations for rural locations should focus on improving local care options whilst considering resource limitation, such as telehealth clinics.

**Supplementary Information:**

The online version contains supplementary material available at 10.1186/s12884-024-07093-8.

## Background

Diabetes in pregnancy (DIP) is a condition that requires intensive management by those diagnosed throughout their pregnancy [1–3]. For the purposes of this research, DIP refers to gestational diabetes mellitus (GDM) as well as those with pre-existing type 1 or type 2 diabetes in pregnancy [5, 6]. Effective management of DIP reduces the risk of adverse events such as increased birthweight and increased risk of neonatal intensive care admissions [4, 5]. DIP management involves daily blood glucose monitoring, dietary changes and the commencement of medication, as clinically indicated [1–3, 6]. To support these changes, education and monitoring by health professionals such as diabetes educators and medical nutrition therapy by a dietitians is recommended [2, 6]. As a result the process of managing DIP can result in significant treatment burden on the individual [7, 8]. People who have experienced DIP report that the combination of blood glucose monitoring, dietary changes and attending frequent healthcare appointments is time consuming and burdensome [7]. It should be noted that for the purposes of this research, terms such as ‘women and people’ or ‘people with DIP’ have been used as gender inclusive and gender-neutral language. This aligns with terminology suggested in recent literature [9, 10]. 

Limited access to services for those in rural areas contributes to worse health outcomes for people living in rural communities [11]. In economically developed countries with comparable rural healthcare disparities to Australia, an increased risk of adverse infant outcomes, such as congenital abnormalities and perinatal mortality, has been reported in rural compared to metropolitan areas, for those with DIP [12, 13]. Additionally, a state-wide study in Australia found infants from individuals with pre-existing DIP in rural Australia experienced worse health outcomes compared to their metropolitan counterparts [14]. A recent national report has described the triple healthcare disadvantage experienced by rural Australians consisting of poorer social determinants of health, poor service availability and higher cost of access and delivery [15]. These issues are reflected in rural maternity care services in Australia, with a lack of antenatal and postnatal services, financial burden and transport difficulties reported by women giving birth in rural Tasmania [16]. 

Research into the experiences of people with DIP globally has predominately been undertaken using qualitative methods such as interviews or focus groups [7, 8]. A systematic review of women’s experiences of a gestational diabetes mellitus (GDM) diagnosis published in 2020 identified 18 studies conducted in Europe, 10 in Australia and nine in North America, with the remaining four in South America and Asia, all with an interview or focus group component [7]. Only two of the 41 included studies focused on individuals in rural areas [7], one in China [17] and the other in the USA [18]. The review highlighted the importance of health professionals providing evidence based care and providing sufficient ongoing support, and understanding the impact of a diagnosis of GDM for individuals [7]. 

Two further Australian studies using qualitative methods, one each from 2009 to 2014 were also identified [19, 20]. One small study (*n* = 7 participants) reported experiences of people with type 1 DIP through individual interviews [19]. This rural-based study [19] along with the 2014 paper exploring experiences with gestational diabetes mellitus in a survey format [20] reported similar key themes related to the psychological impact of GDM, the need for health professional support and the burden of management, and congruent with the existing international literature [7, 20] However, there was no comparison of metropolitan and rural respondents.

There is a lack of research into the experiences of individuals with DIP living in rural areas of Australia, despite the additional challenges in accessing care and the potential for worse health outcomes for this group. Additionally, no studies to date have compared metropolitan and rural experiences of women and people with DIP. Therefore, the primary aim of this study was to explore ways to improve healthcare delivery from the perspectives of those who had previously been diagnosed with DIP in Australia. The secondary aim was to compare a metropolitan and rural responses.

## Methods

A cross-sectional survey of people who had recently experienced DIP in Australia was undertaken, as part of a larger mixed methods study. The survey included 26 multiple choice questions and three open ended questions. Open ended questions provided a space for unlimited free text to allow participants to provide detailed responses. Survey questions can be found in supplementary file 1. This current paper focuses on responses to the open-ended question: ‘If anything, what would you want changed about the care you received by health professionals throughout your experience with diabetes in pregnancy?’. The online survey and data collection were completed via REDCap (HMRI REDCap, version 13) [21, 22], a web-based application designed for secure research focused data collection.

The University of Newcastle Human Research Committee provided ethics approval for this research (H-2021-0425). Eligible survey participants included individuals over 18 years who had experienced any form of DIP, including pre-existing type 1 diabetes or type 2 diabetes or GDM in the previous two years and received their health care in Australia. Individuals were considered ineligible to complete the survey if they were currently pregnant.

Diabetes Australia’s National Diabetes Services Scheme (NDSS) is free for any Australian diagnosed with diabetes to join, with the aim of the program to provide support and subsidised services [23]. Those registered are able to opt-in to receiving invitations to participate in diabetes-related research. The NDSS sent Invitations on behalf of the researchers, with emails sent to a target group of potentially eligible individuals. Researchers did not have access to the NDSS registrant information. The survey was open throughout April and May of 2023.

Demographic data collected from participants included age, location of residence during their most recent DIP experience, ethnicity, Aboriginal and/or Torres Strait Islander status, place of birth and education level. Results from all demographics questions have been reported elsewhere [paper under review]. Participants were categorised as metropolitan or rural residents using the Modified Monash Model (MMM) classification system based on their reported location of residence [24]. This classification system categorises locations based on population size and remoteness [24]. Respondent location of residence was dichotomised into either ‘metropolitan’ (all MMM1) or ‘rural’ (MMM2-7) and their open ended responses were grouped accordingly.

**Fig. 1:**
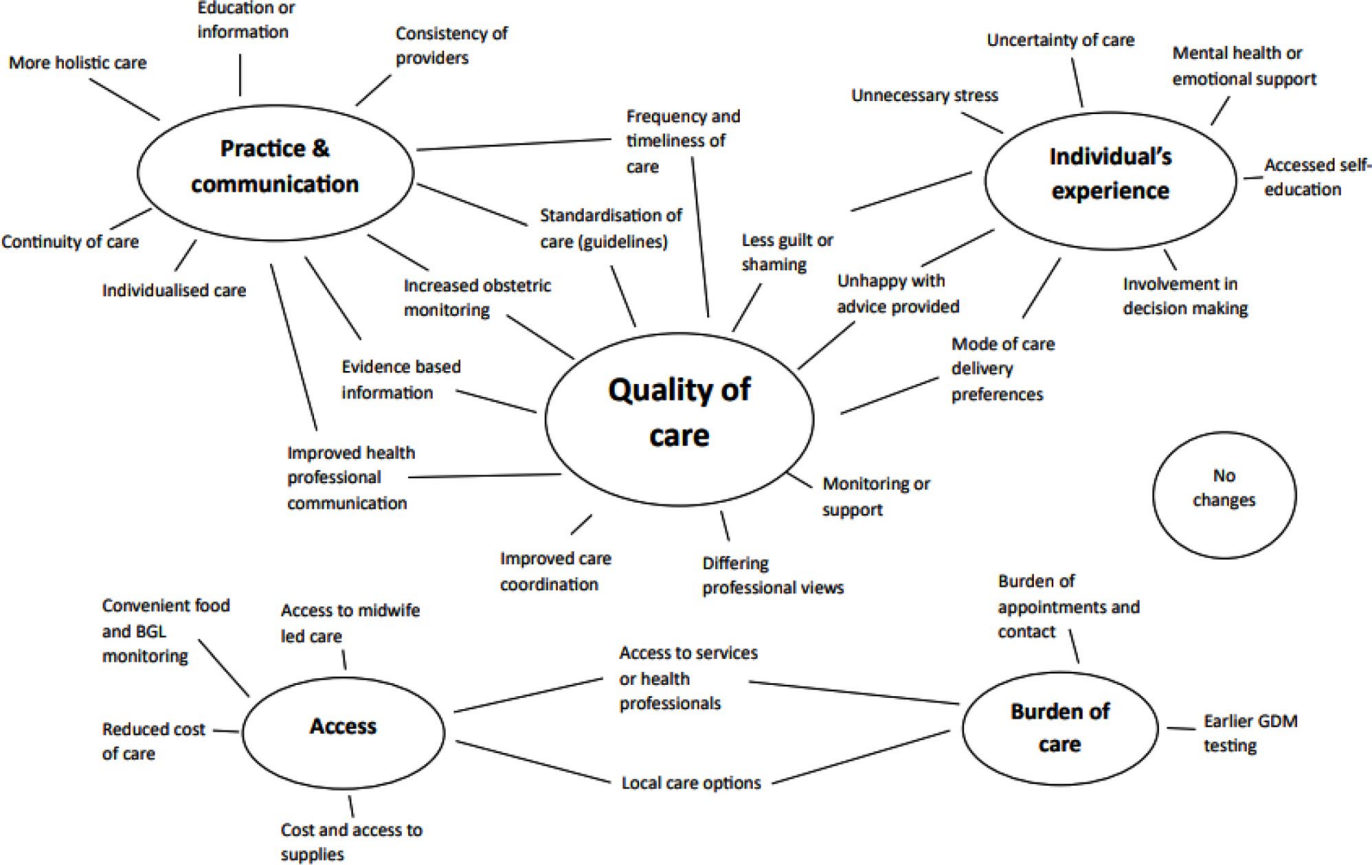
Identified codes linked to relevant themes

Qualitative content analysis was used for interpretation of the open-ended survey question responses [25, 26]. This type of analysis offers a flexible, systematic approach to interpreting qualitative data that is suited to datasets containing a large number of responses [27, 28]. An inductive approach was used [28], whereby initial codes were created by one researcher (EP) using NVivo Pro 12 [29] to organise the data. These initial codes were discussed with other team members (LB and SH) in an iterative process conducted over several meetings, where quotes were organised and re-organised under codes until the team came to a consensus with the codes and supportive quotes. The same process was used to establish overarching themes with each code linking to one or more themes (see Fig. 1).

After the codes and themes were established, the total number of quotes assigned to each code was calculated. Code frequency was also separated by metropolitan and rural response frequency. Themes and underlying codes with representative quotes are described. A description of key demographic and health data for respondents from key survey questions is provided for context. Each illustrative quote is labelled with the participants rural (MMM2-7) or metropolitan (MMM1) residency.

## Results

### Description of survey respondents

A total of 668 individuals responded to the survey, with 409 of these participants providing a written response to the qualitative research question reported in this paper. From these, 71.6% of respondents were from a metropolitan area, and 27.6% from a rural area (12.0% MMM2, 9.6% MMM3-4, 6.0% MMM5-7). Respondents were distributed across all states and territories, with the majority of respondents from New South Wales (37.2%), Queensland (20.8%) or Victoria (21.3%). A total of 3.4% of respondents identified as Aboriginal and/or Torres Strait Islander. Most respondents identified as Caucasian ethnicity (71.1%), with 13.9% Asian, 6.4% European and 7.3% as other ethnicities. Respondents reported high levels of education, with 72.6% reporting completing of a bachelor or post-graduate degree. A summary of demographics is available in Table 1.

**Table 1:** Demographics of respondents to qualitative survey question (*n* = 409)

Modified Monash Model (MMM)	*n* (%)
0	3 (0.7%)
1	293 (71.6%)
2	49 (12.0%)
3	24 (5.9%)
4	15 (3.7%)
5	23 (5.6%)
6	1 (0.2%)
7	1 (0.2%)
State/ territory	**n (%)**
NSW	152 (37.2%)
QLD	85 (20.8%)
VIC	87 (21.3%)
WA	27 (6.6%)
SA	27 (6.6%)
NT	4 (1.0%)
TAS	6 (1.5%)
ACT	19 (4.6%)
No response	2 (0.5%)
Indigenous status	**n (%)**
Yes	14 (3.4%)
No	390 (95.4%)
Prefer not to say	5 (1.2%)
Ethnicity	**n (%)**
Caucasian	291 (71.1%)
Asian	57 (13.9%)
European	26 (6.4%)
Other	30 (7.3%)
Polynesian or Maori	5 (1.2%)
Level of education	**n (%)**
Secondary education year 9 or below	5 (1.2%)
Secondary education years 10–12	21 (5.1%)
Certificate III or IV	38 (9.3%)
Diploma or advanced diploma	46 (11.2%)
Bachelor’s degree	178 (43.5%)
Post graduate qualifications (Masters)	119 (29.1%)
Other	2 (0.5%)

The majority of both metropolitan (77.5%) and rural (76.6%) respondents had only experienced one pregnancy with a diagnosis of diabetes. The most common treatment methods for glycaemic control were diet and lifestyle (50.9% metropolitan, 44.7% rural), followed by insulin (36.2% metropolitan, 36.6% rural) and then oral hypoglycaemic medication (e.g. metformin) (11.4% metropolitan, 16.7% rural).

### Qualitative data

A total of 15 different respondents are included in the quotes below from the themes developed using the data from 409 individuals who provided a typed response to the open-ended question.

### Quality of care

The overarching theme quality of care connected to 11 of the 31 codes established from participant responses. Participant quotes have been presented exactly as provided in the survey responses. People with previous DIP reported wanting differences in the way their care was delivered, specifically relating to the logistics of this care delivery.

Mode of care delivery preferences - *“I would have liked to have talked face to face with diabetes educators more often. only had telehealth appointment and email correspondence.”* – rural respondent.

Monitoring or support - *“More support*,* particularly around taking insulin would have been good. I didn’t feel confident in what I was doing most of the time”* – rural respondent.

Additionally, those with previous DIP found inconsistencies in their care delivery an issue, both relating to the advice provided by individual health professionals, and variation in the treatment guidelines by location.

Differing professional views *- “There were times I was given conflicting information within the same day by the health professionals*,* which was disheartening and frustrating and I felt like I was treated focused solely on my BMI rather than more wholistically”* – metropolitan respondent.

Standardisation of care (guidelines) - *“The difference in criteria between states is also very confusing for patients e.g. my 2-hour readings needed to be under 6.7[mmol/L] but other states were either stricter or more lenient.”* – metropolitan respondent.

Individuals with previous DIP reported feelings of guilt, fear and shame regarding communication from certain health professionals. Participants reported feeling they were being shamed as a result of their body size and pressured to follow advice provided due to the strong emphasis of potential adverse outcomes for the baby.

Less guilt or shaming - *“If I could change one thing about the care I received would be informed and empowered to make my own decisions that are right for my baby and I. Rather than doing things out of ‘fear’ and hospital policies.”* – rural respondent.

## Practice & communication

People with previous DIP reported wanting changes in their care relating to several aspects of health professional practice and communication. Participants discussed having generally improved communication from health professionals, as well as more education and information provided as part of their care.

Improved health professional communication - *“More contact from professionals I feel like I was forgotten about and left to figure it out on my own.”* – rural respondent.

Education or information - *“Some more hands on guidance for helping with the diet. With the educators they just wanted my numbers and there wasn’t much in terms of actual assistance”* – metropolitan respondent.

Respondents also highlighted the need for more individualised care delivery by their health professional providers.

Individualised care - *“It was a group setting and the approach was very one way fits all style. It felt somewhat disconnected and I didn’t feel individualised for treatment options”* – metropolitan respondent.

Changes in the frequency of appointments and limiting the delay between diagnosis and initial appointments were also discussed.

Frequency or timeliness of care - *“The space between being diagnosed and actually receiving treatment was longer than I’d hoped (approx. 4 weeks) because we had to wait for appointments at set scheduled times.”* – rural respondent.

Consistency of providers - *“I never saw the same diabetes educator or obstetrician twice*,* I was continually seeing new people and having to re explain my story which made the whole pregnancy very difficult. I would have appreciated some consistency in care.”* – rural respondent.

## Individual’s experience

Codes under the individual’s experience focused on the impact of care on the individual with previous DIP. Respondents reported the uncertainty of care to be an issue impacting their experience. The consequences of this uncertainty for rural individuals were exacerbated compared to metropolitan, as described by the below participant.

Uncertainty of care - *“From 26 weeks − 38 weeks I did not know if I was birthing in Broken Hill/Adelaide or Sydney. The midwives kept telling me that the OB/GYN would make that decision at the next appointment*,* but each time the locum doctor would say ‘we’ll make the decision next time/the next doctor can make that decision’. I found that incredibly stressful (to the point I was referred to the mental health team at broken hill hospital)”* – rural respondent.

Particular communication practices by healthcare providers were reported to cause unnecessary stress for respondents.

Unnecessary stress *- “The constant fear mongering because I was going to have a ‘big baby’ was stressful and provided unnecessary pressure and fear in an already difficult and stressful situation.”* – metropolitan respondent.

### Access

Codes relating to access discussed access to general care, access to specific health professionals and access to supplies. Participants cited the importance of maintaining midwives in their care model, which was not always an option provided once diagnosed with GDM.

Access to midwife led care - *“I also wish there had been more options for model of care - I was only able to go through the complex care clinic at the hospital and would have preferred a midwife-led mod”* – metropolitan respondent.

Having increased access to local care options was a change requested by primarily rural respondents.

Local care options *- “Initially there was no diabetes educator in broken hill & I would have had to travel to Adelaide to get that help”* – rural respondent.

## Burden of care

The burden of care theme conveys the load placed on the individual due to accessing the care required to manage their condition. Limited appointment access resulted in increased burden of care, as individuals would need to book appointments well in advance, or change their schedule based on health professional availability.

Access to services or health professionals - *“Need more professionals trained as there were times they were booked out and I missed appointments or was going in every week to make up an appointment”* – metropolitan respondent.

Frequent appointments were reported to increase the care burden for individuals with previous DIP.

Burden of appointments and contact - *“I wish there was an online screening test to reduce the amount of appointments*,* particularly the telehealth appointments. Telehealth alternated with the physical appointments.”* – metropolitan respondent.

Figure 1 depicts the codes and overarching themes developed through the content analysis of participant quotes. The overarching themes have been mapped and connected to the underlying codes. The five themes consist of (i) practice and communication, (ii) individual’s experience, (iii) quality of care, (iv) access and (v) burden of care.

**Fig. 2:**
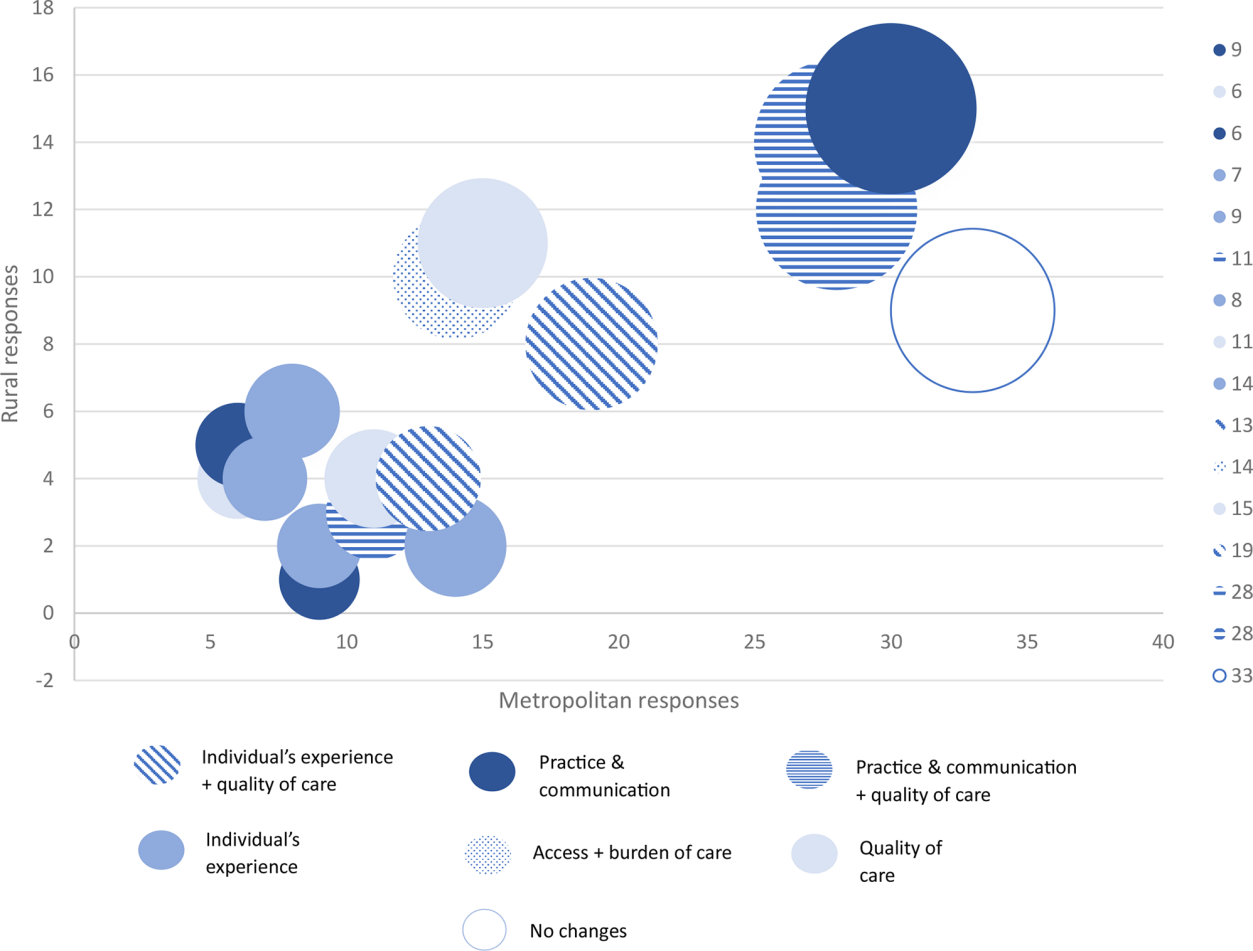
Metropolitan and rural respondents frequency quotes assigned to codes with 10 or more quotes

Figure 2 shows the number of quotes assigned to codes from metropolitan and rural participants. The codes with the largest number of assigned quotes regardless of location were *education or information* (*n* = 45), *frequency and timeliness of care* (*n* = 42), *no changes* (*n* = 42) and *improved health professional communication* (*n* = 40) (see supplementary file 2). Several codes were assigned to metropolitan respondents quotes only, including *cost and access to supplies*, *convenient food and BGL monitoring*, *evidence-based care*, *increased obstetric monitoring* and more holistic care. *Local care options* was the only code that contained more rural quotes (*n* = 4) compared to metropolitan (*n* = 1). Further information regarding the breakdown of code assignments between metropolitan and rural responses can be found in supplementary file 2.

## Discussion

To the authors’ knowledge, this is the first qualitative analysis of survey data from a mixed methods Australian study comparing the perspectives of women and pregnant people in metropolitan and rural areas. Additionally, previous papers from Australia and other economically comparable countries have focussed on the individual’s general experience in dealing with DIP diagnosis and journey, rather than experiences of and areas for improvement in healthcare delivery [7, 20, 30]. With the exception of a qualitative survey published in 2014 [20], previous Australian qualitative research has utilised interviews or focus groups [19, 30], with small participant numbers or a focus on a specific service. This paper provides patient perspectives on how to improve care delivery from a large sample of those who have experienced DIP, from services across diverse rural and metropolitan areas of Australia.

Notably, several codes were represented by quotes from metropolitan responses only. These included *cost and access to supplies*, *convenient food and BGL monitoring*, *evidence-based care*, *increased obstetric monitoring* and *more holistic care*. The metropolitan representation of quotes for these codes may be due to the 73% of respondents residing in a metropolitan location, or it may be because rural respondents found other issues a high priority, such as local access to services. Interestingly, convenience and access, factors often considered more problematic for those in rural areas were not highlighted in this sample. Conversely *local care options* was the only code that contained more rural quotes than metropolitan. This likely reflects the issues of poor access to services, which disproportionately affects rural residents in Australia due to low population density, geographic spread and limited infrastructure compared to metropolitan areas [11, 31]. 

While some codes were mentioned more frequently by metropolitan or rural respondents, the major codes were more evenly distributed from both metropolitan and rural respondents. Though both Australian and international research is lacking, other studies focusing on rural respondents [18, 19] found similar themes to those studies that did not differentiate respondents by location [8, 20, 32, 33]. Struggling with the diagnosis of DIP and associated feelings such as fear or guilt, as well as communication with health professionals were key themes in these papers [8, 18, 19, 32, 33]. This suggests that though there may be specific issues for those residing in rural compare to metropolitan locations, the most prevalent issues are occurring regardless of location.

The most frequently occurring codes across both metropolitan and rural responses were *education or information*, *frequency and timeliness of care* and *improved health professional communication*. Each of these codes had a strong representation of quotes from both metropolitan and rural respondents. These three codes were all connected to the overarching theme *practice and communication*, highlighting the importance of communication is as part of the care experience for individuals with DIP. Health professional communication has been identified as a key part of the individual with DIP’s experience in previous qualitative research [7, 19, 20]. However, it is important to note that previous qualitative papers did not specifically focus on aspects of care delivery that could be improved, and instead themes were based on the individual’s experience coping with the diagnosis [20, 30]. 

Recommendations supported by previous research and this current study to improve the experience for women and pregnant people with DIP is for healthcare services to develop systems that ensure timely care is provided in accordance with current guidelines [1, 2, 6]. Similarly, establishing a national audit of DIP care in Australia would assist in improving care delivery for those with DIP, particularly those experiencing difficulties accessing healthcare such as those residing in rural Australia [34]. Current consensus based national guidelines exist for people with pre-existing DIP [6], however Australian GDM management guidelines vary by state and/or health district [2, 3]. A national management guideline would assist with improving consistency of education, information and communication by health professionals. Improvements in communication can occur across two primary domains; multidisciplinary health professional communication between health professionals, and health professional communication with individuals with DIP. For rural services, a priority is ensuring that individuals with DIP have the option to access their care locally, tailoring strategies to achieve this according to the resources available in a specific region [11]. From the current study, addressing workforce shortages and offering telehealth or mixed service delivery options are possible strategies to improve local care access. Additional research seeking perspectives from other involved stakeholder groups is required to ensure realistic recommendations are developed given workplace issues and service delivery challenges.

A strength of the current study is the large number of respondents participating in the survey from a diverse range of locations and backgrounds across Australia, which provided a breadth of open-ended responses for qualitative content analysis. This also allowed for comparison between rural and metropolitan respondents to explore any difference in their perspectives. A limitation is that researchers were unable to compare frequency of codes with the number of responses due to the nature of the qualitative coding process. Another limitation is the inability to calculate a response rate, as the total number of potential participants reached is unknown. Additionally, respondents were highly educated and required computer or smartphone access as well as internet access to complete the survey. This may have limited the overall diversity of the sample.

## Conclusion

The current study highlighted potential areas for healthcare delivery improvement from the perspectives of women and people with previous DIP. Both metropolitan and rural respondents indicated education, frequency and timeliness of care delivery and health professional communication were key areas for change to occur. Rural respondents prioritised access to local care options, whereas metropolitan respondents requested access to supplies, convenient monitoring and access to holistic care. Recommendations for change from this study include improving health professional communication and timely access to services, as priorities for improving the experience of care delivery for women and people with previous DIP.

## Electronic supplementary material

Below is the link to the electronic supplementary material.

Supplementary Material 1

Supplementary Material 2

## Data Availability

The datasets used and/or analysed during the current study are available from the corresponding author on reasonable request.

## Abbreviations

DIP: diabetes in pregnancy
GDM: gestational diabetes mellitus
NDSS: National Diabetes Services Scheme
MMM: Modified Monash Model
