# Supplementary material for: Exploring the experiences of women and people with diabetes in pregnancy in metropolitan and rural Australia: a national survey
Source: BMC Pregnancy Childbirth. 2025 Jan 8;25:16. doi: 10.1186/s12884-024-07093-8 (PMC11708241; doi:10.1186/s12884-024-07093-8)
Supplement: Supplementary file 1 — Supplementary Material 1 [file 12884_2024_7093_MOESM1_ESM.pdf]

Women with previous diabetes in pregnancy survey

Thank you for clicking on the link for this survey.

A team of researchers from the University of Newcastle would like your help to understand and compare current metropolitan and rural healthcare delivery for women with diabetes in pregnancy across Australia.

We are seeking women who recently (within the last 2 years) experienced diabetes in pregnancy, inclusive of gestational diabetes mellitus, type 1 or type 2 diabetes mellitus in pregnancy, who were living in Australia at the time of their diagnosis and treatment.

If you agree to participate, you will be asked to complete a survey asking questions regarding your experiences with the treatment you received for your diabetes in pregnancy in Australia. Once you have completed the survey, you will be asked if you are willing to participate in an interview as an extension of this research.

This project has been approved by the University's Human Research Ethics Committee, Approval No. H- [insert the protocol reference number which will be identified in the written acknowledgement of your application].

Should you have concerns about your rights as a participant in this research, or you have a complaint about the manner in which the research is conducted, it may be given to the researcher, or, if an independent person is preferred, to the Human Research Ethics Officer, Research & Innovation Services, The University of Newcastle, University Drive, Callaghan NSW 2308, Australia, telephone (02) 4921 6333, email Human-Ethics@newcastle.edu.au.

If you would like to know more about the survey, click on the link below labelled 'participant information statement - women'. Please read the participant information statement before completing the survey.

[Attachment: "PIS women 2022_01_25.doc"]

Do you wish to participate in this study?<br><br>(Selecting 'yes' will indicate you have read the participant information statement and you consent to your responses being used for research)	◯ Yes<br>◯ No

The following questions are screening questions to determine your eligibility to complete the survey.

Have you experienced diabetes in pregnancy (gestational diabetes, type 1 or type 2 diabetes in pregnancy) in the last 2 years?	◯ Yes<br>◯ No
Were you living in Australia at the time of this pregnancy?	◯ Yes<br>◯ No
Were you over the age of 18 when you experienced this pregnancy?	◯ Yes<br>◯ No
Are you currently pregnant?	◯ Yes<br>◯ No

This survey asks questions regarding your experiences with diabetes in pregnancy (gestational diabetes, Type 1 or Type 2 diabetes in pregnancy). Section A: Demographic information

The following questions are to find out about you and your background.

When thinking about your most recent experience of diabetes in pregnancy, which type of diabetes in pregnancy did you experience?	○ Gestational diabetes mellitus<br>○ Type 1 diabetes in pregnancy<br>○ Type 2 diabetes in pregnancy<br>○ Unsure
How old are you (in years) as of today?	○ 18-24<br>○ 25-35<br>○ 36-45<br>○ 46+
Were you born in Australia?	○ Yes<br>○ No
If no, what country were you born in?	__________________________________
Do you identify as Aboriginal or Torres Strait Islander?	○ Yes, Aboriginal<br>○ Yes, Torres Strait Islander<br>○ Yes, both Aboriginal and Torres Strait Islander<br>○ No<br>○ Prefer not to answer
What is your ethnicity?	○ Caucasian<br>○ Asian<br>○ European<br>○ Polynesian or Maori<br>○ Other (please specify) ______
What is the highest level of education you have completed?	○ Secondary education - Year 9 or below<br>○ Secondary education - Years 10-12<br>○ Certificate III or IV<br>○ Diploma or Advanced diploma<br>○ Bachelor's Degree<br>○ Postgraduate qualifications (i.e. Masters)<br>○ Other (please specify) ______
How many times have you given birth (gestation 20 weeks or more)?<br><br>Please note: you can contact a mental health crisis line (Lifeline 131114) or seek care from your local general practitioner should you wish to seek support regarding any of the issues raised within the survey.	○ 1<br>○ 2<br>○ 3<br>○ 4<br>○ 5 or more<br>○ Prefer not to say
In how many pregnancies have you experienced diabetes in pregnancy, including your most recent pregnancy?	○ 1<br>○ 2<br>○ 3<br>○ 4<br>○ 5 or more

The following questions are referring to your MOST RECENT experience with diabetes in pregnancy.

Section B: Diagnosis of diabetes in pregnancy

The following questions relate to your diagnosis with diabetes in pregnancy. If you experienced type 1 or type 2 diabetes in pregnancy, questions relate to your diagnosis before becoming pregnant.

How many weeks pregnant were you when you were diagnosed with gestational diabetes? (in your most recent pregnancy with diabetes)

- ◯ Less than 13 weeks
- ◯ 13-23 weeks
- ◯ 24-28 weeks
- ◯ Greater than 28 weeks
- ◯ Unsure

How long had you been diagnosed with diabetes before your most recent pregnancy?

- ◯ < 1 year
- ◯ 1 - 2 years
- ◯ 2 - 5 years
- ◯ 5 - 10 years
- ◯ > 10 years

Did you see a health professional for advice pre-conception for your diabetes?

- ◯ Yes
- ◯ No
- ◯ Unsure

Section C: Experiences of management of diabetes in pregnancy

The following questions relate to the management of your diabetes in pregnancy.

Where were you living at the time you experienced diabetes in pregnancy? (please provide town or suburb and postcode) __________________________________

Which health professionals did you access services from as part of your treatment and where did you see these health professionals?

	I saw this health professional my home town	I had to travel to see this health professional	I used telehealth to see this health professional (e.g. phone, internet)	I did not see this health professional
Diabetes educator	○	○	○	○
Dietitian	○	○	○	○
Endocrinologist	○	○	○	○
Obstetrician	○	○	○	○
Nurse/ midwife	○	○	○	○
Physiotherapist/ exercise physiologist	○	○	○	○
General practitioner	○	○	○	○

What is the maximum distance you had to travel to see any of these health professionals? (in kilometres) ________________________________

How many weeks pregnant were you when you first saw a health professional to manage your diabetes in pregnancy? ________________________________

How often did you have contact with the health professionals you saw throughout your pregnancy?

	Weekly or more often	Fortnightly	Monthly	Only saw once/ ad hoc	Unsure	Not seen
Diabetes educator	◯	◯	◯	◯	◯	◯
Dietitian	◯	◯	◯	◯	◯	◯
Endocrinologist	◯	◯	◯	◯	◯	◯
Obstetrician	◯	◯	◯	◯	◯	◯
Nurse/ midwife	◯	◯	◯	◯	◯	◯
Physiotherapist/ exercise physiologist	◯	◯	◯	◯	◯	◯
General practitioner	◯	◯	◯	◯	◯	◯

How did you manage your diabetes in pregnancy? (choose all that apply)

- ☐ Diet and lifestyle only
- ☐ Insulin
- ☐ Metformin (oral/tablet medication)
- ☐ Unsure
- ☐ Other (please specify) ______

If anything, what would you want changed about the care you received by health professionals throughout your experience with diabetes in pregnancy?

____

What type of birth did you have?

- ◯ Vaginal birth
- ◯ Vaginal birth (induced labour)
- ◯ Assisted vaginal birth (with vacuum extraction or forceps)
- ◯ Caesarean section before going into labour (planned)
- ◯ Caesarean section after going into labour (emergency)
- ◯ Other (please specify)
- ◯ Unsure/ prefer not to say

Section D: Postpartum diabetes in pregnancy management The following questions relate to your experience with postpartum diabetes in pregnancy management.

Did you receive a postpartum follow up glucose test? (this may have occurred 6-12 weeks after your pregnancy)	☐ Yes<br>☐ No<br>☐ Unsure
If yes, who performed this test?	◯ General practitioner<br>◯ Diabetes educator<br>◯ Unsure<br>◯ Other (please specify) ______
How satisfied were you with the overall care you received from health professionals throughout your experiences with diabetes in pregnancy?	◯ Very satisfied<br>◯ Somewhat satisfied<br>◯ Neutral<br>◯ Somewhat unsatisfied<br>◯ Very unsatisfied
How satisfied were you with the communication between the health professionals caring for you throughout your experience with diabetes in pregnancy?	◯ Very satisfied<br>◯ Somewhat satisfied<br>◯ Neutral<br>◯ Somewhat unsatisfied<br>◯ Very unsatisfied
If very satisfied, what worked well?	______________________________
What aspects of the management of your diabetes in pregnancy could have been improved or changed?	______________________________
