# Supplementary material for: Exploring the experiences of women and people with diabetes in pregnancy in metropolitan and rural Australia: a national survey
Source: BMC Pregnancy Childbirth. 2025 Jan 8;25:16. doi: 10.1186/s12884-024-07093-8 (PMC11708241; doi:10.1186/s12884-024-07093-8)
Supplement: Supplementary file 2 — Supplementary Material 2 [file 12884_2024_7093_MOESM2_ESM.docx]

|  | Overarching themes | | | | | | | | | | | | | | | | |
| --- | --- | --- | --- | --- | --- | --- | --- | --- | --- | --- | --- | --- | --- | --- | --- | --- | --- |
|  | **Practice and communication** | | | **Individual’s experience** | | | | **Quality of care** | | | **Individual’s experience + quality of care** | | **Practice & communication + quality of care** | | **Access + burden of care** | | **No changes** |
| Codes | **Consistency of providers** | **Education or information** | **Individualised care** | **Involvement in decision making** | **Unnecessary stress** | **Mental health or emotional support** | **Accessed self-education** | **Monitoring or support** | **Differing professional views** | **Improved care coordination** | **Less guilt or shaming** | **Mode of care delivery preferences** | **Frequency and timeliness of care** | **Improved health professional communication** | **Standardisation of care (guidelines)** | **Access to services or health professionals** | **No changes** |
| Metropolitan  respondent | 6 | 30 | 9 | 7 | 8 | 9 | 14 | 6 | 15 | 11 | 19 | 13 | 28 | 28 | 11 | 14 | 33 |
| Rural respondent | 5 | 15 | 1 | 4 | 6 | 2 | 2 | 4 | 11 | 4 | 8 | 4 | 14 | 12 | 3 | 10 | 9 |
| Total | 11 | 45 | 10 | 11 | 14 | 11 | 16 | 10 | 26 | 15 | 27 | 17 | 42 | 40 | 14 | 24 | 42 |

**Supplementary file 1: frequency of codes comparing metropolitan and rural respondents**
